# Case report: Hyperosmolar hyperglycemic syndrome secondary to PEG-asparaginase-induced hypertriglyceridemia and pancreatitis

**DOI:** 10.3389/fonc.2022.1094964

**Published:** 2023-01-19

**Authors:** Todd William Mudd, Ashley Danielle Fox, Mark Ghaly, Amany Keruakous

**Affiliations:** ^1^ Medical College of Georgia, Augusta, GA, United States; ^2^ Department of Internal Medicine, Medical College of Georgia at Augusta University, Augusta, GA, United States; ^3^ Georgia Southern University, Savannah, GA, United States; ^4^ Department of Hematology and Oncology, Georgia Cancer Center, Augusta, GA, United States

**Keywords:** ALL, PEG-asparaginase, toxicity, hyperosmolar hyperglycemic syndrome, pancreatitis

## Abstract

Pegylated (PEG)-asparaginase is an established treatment for acute lymphoblastic leukemias that exhibits an antitumor effect by depleting asparagine, an amino acid essential for leukemia cell protein synthesis. Pancreatitis with hypertriglyceridemia is a well-established toxidrome associated with PEG-asparaginase. However, impaired pancreatic synthetic function and hormone release have rarely been reported as a result of PEG-asparaginase pancreatitis. In this report, we present a 22-year-old woman recently diagnosed with T-acute lymphoblastic leukemia (T-ALL), who presented to the hospital with progressive weakness, confusion, blurry vision, hallucinations, and abdominal pain after induction treatment with daunorubicin, vincristine, PEG-asparaginase, and dexamethasone following the AYA protocol. She was found to have hypertriglyceridemia, acute pancreatitis, and hyperosmolar hyperglycemic syndrome. While pancreatitis and hypertriglyceridemia are commonly reported side effects of PEG-asparaginase, HHS related to these conditions has been sparsely reported. Providers should maintain awareness of this association and consider routine serial glucose monitoring of patients receiving PEG-asparaginase.

## Background

l-Asparaginase is an *Escherichia coli*- and *Erwinia chrysanthemi*-derived enzyme that catalyzes the breakdown of the essential amino acid asparagine into aspartate and ammonia. Studies of l-asparaginase activity in leukemia cells have found that the exhibition of an antineoplastic effect is caused by depleting cancer cells of the essential amino acid asparagine, leading to impaired protein synthesis and apoptosis, as tumor cells lose asparagine synthetase activity during neoplastic transformation (1). The use of the pegylated form of l-asparaginase (PEG-asparaginase) has historically been an underpinning component in treatment protocols for childhood acute lymphoblastic leukemia (ALL).

Significant superiority in overall survival (OS) of childhood ALL compared to adult populations was attributed to multiple factors, including genetic differences between adult and pediatric ALL and differences in the chemotherapy agents and dose intensities for the two populations, including the use of PEG-asparaginase in pediatric regimens (2). Studies comparing the treatment of adolescent and young adult (AYA) patients with pediatric *vs*. adult ALL regimens found that the pediatric regimens resulted in improved survival outcomes, supporting the idea that treatment regimen differences drove divergence between adult and pediatric ALL outcomes (2–5). These findings resulted in updates to adult ALL treatment protocols, including the addition of PEG-asparaginase (6).

While these updated ALL treatment protocols have improved outcomes in adults, they have also introduced a range of unique side effects associated with PEG-asparaginase. Common adverse effects of PEG-asparaginase include hepatotoxicities such as increased AST/ALT and hyperbilirubinemia, hypertriglyceridemia, venous thromboembolism (VTE), and pancreatitis (7, 8). While PEG-asparaginase-related pancreatitis has been well characterized, rare cases of additional endocrine complications, including diabetic ketoacidosis (DKA) and hyperosmolar hyperglycemic state (HHS), have been reported (9–13). Although there have been cases of combined DKA and PEG-asparaginase-associated pancreatitis (AAP), the reported case of HHS related to PEG-asparaginase occurred in the absence of AAP. Here, we present a case of a 22-year-old woman who experienced AAP and HHS, a previously unreported toxicity combination related to PEG-asparaginase treatment.

Written informed consent was obtained from the patient for the publication of any potentially identifiable images or data included in this article.

## Case presentation

A 22-year-old woman with no past medical history was diagnosed with T-cell ALL (CD3/8+, MPO/TdT negative) in May 2022 after experiencing several months of diffuse lymphadenopathy, early satiety, abdominal distention, generalized weakness, and malaise. Her initial workup included an MRI that showed leptomeningeal enhancement, a negative lumbar puncture, and a bone marrow biopsy that revealed an abnormal clonal population with a gain of one to two copies of *IGH* and a gain of *CMYC*, p16, 9q21, BCR, and ABL. She was started on induction therapy on 6/16/22 with an AYA regimen including intrathecal cytarabine/methotrexate, daunorubicin, vincristine, dexamethasone, and PEG-asparaginase (Children’s Oncology Group protocol AALL 1231) with a plan for an eventual allogeneic stem cell transplant (14).

Initially, she tolerated her regimen while experiencing neuropathy, tremors, gastritis, and low energy levels, with monitoring labs showing mild hypertriglyceridemia and hyperglycemia (**Table 1**). She had been initiated on metformin twice daily for her hyperglycemia in the outpatient setting. Just a few days after the completion of her induction treatment, the patient was brought into the hospital by her family with progressive confusion, blurry vision, hallucinations, and abdominal pain that began days prior. She had received two doses of 2,500 U/m^2^ of PEG-asparaginase, with her most recent dose administered 7 days prior to her presentation in the ED. Per the history provided by her mother, she additionally had decreased oral intake and encountered worsening weakness in addition to her other symptoms. Upon arrival, she was hypotensive with BPs in the 100s/80s, tachycardic to 120s, and acutely altered with severe abdominal pain on exam. Pertinent lab findings on presentation included an elevated serum creatinine of 2.15 mg/dl (significantly above the patient’s baseline), blood glucose 2,035 mg/dl, serum osmolality 430 mOsm/kg, serum sodium 130 mEq/L, serum bicarbonate of 14 mg/dl, lipase of 986 U/L, and triglycerides of 1,727 mg/dl. Urinalysis showed marked glucosuria but was negative for ketones. An arterial blood gas demonstrated a pH of 7.25, partial pressure of carbon dioxide (PaCO_2_) of 18.7 mmHg, and partial pressure of oxygen (PaO_2_) of 136 mmHg.

**Table 1:** Overview of pertinent laboratory data comparing routine labs obtained at a hematology/oncology clinic visit 5–7 days prior to hospital admission to labs obtained in the emergency department on presentation.

	Outpatient monitoring labs	Labs on presentation to ER
Serum creatinine (mg/dl)	0.69	2.15
Serum bicarbonate (mEq/L)	21	14
Blood glucose (mg/dl)	354	2,035
Serum osmolality (normal: 275–295 mOsm/kg)	–	430
Triglyceride (normal: 30–140 mg/dl)	290	1,727
Lipase (normal: 6–51 U/L)	27	986

A CT scan of the abdomen demonstrated mild multifocal pancreatic edema suggestive of acute pancreatitis. The patient was admitted to the intensive care unit and managed for PEG-asparaginase-induced pancreatitis and hyperosmolar hyperglycemic syndrome (HHS) with aggressive intravenous resuscitation, continuous insulin infusion, conservative osmolality adjustment to prevent cerebral edema, and aggressive correction of her other electrolyte abnormalities according to the institutional HHS protocol. On presentation, she was noted to be in hypovolemic shock, requiring vasopressor support with norepinephrine and vasopressin, and acute respiratory insufficiency secondary to altered mentation, requiring intubation for airway protection. She was started on stress-dose hydrocortisone for relative/partial adrenal insufficiency. It was determined that the likely cause of her HHS was related to a combination of dexamethasone-induced insulin resistance and pancreatitis secondary to PEG-asparaginase toxicity.

**Figure 1:**
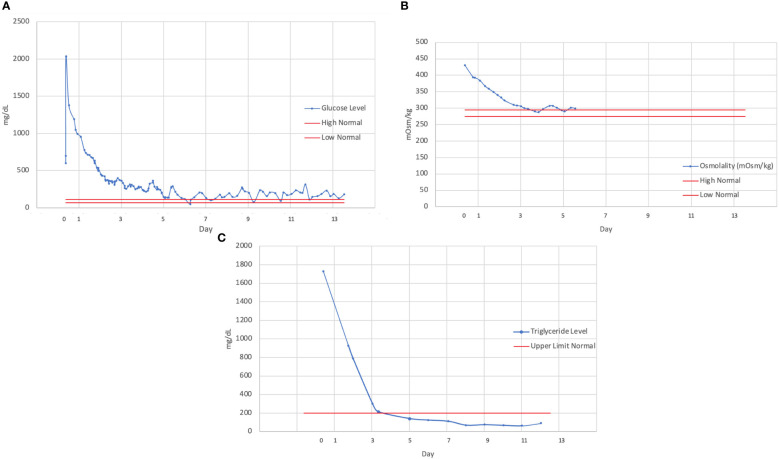
Trend of **(A)** serum glucose, **(B)** osmolality, **(C)** and triglyceride levels over the course of hospitalization, demonstrating a slow rate of correction over several days. Continuous intravenous insulin was discontinued on hospital day 5 (beginning with day of admission as day 0).

The patient experienced improvements in her mental status with the slow correction of hyperglycemia, and she was extubated without complication. Her hypertriglyceridemia and hyperglycemia were both corrected throughout her hospitalization with insulin administration (**Figure 1**). The patient was ultimately transitioned from intravenous insulin to subcutaneous insulin glargine with sliding-scale insulin. She experienced several additional complications throughout her hospitalization, including postintensive care syndrome with severe myopathy, critical illness ileus, and an exacerbation of her chemotherapy-associated neuropathy. After 2 weeks, the patient was discharged from the hospital on a hydrocortisone taper, along with a regimen that included insulin glargine and metformin. She denied any prior history of dyslipidemia or hyperglycemia and did not know of any family history of diabetes or dyslipidemia.

After discharge, the patient continued insulin glargine 5 U QHS and metformin 500 mg BID with good control of her blood glucose levels at subsequent clinic visits. She was first weaned off of her insulin glargine and subsequently off of metformin with good glucose control and continued her bone marrow transplant evaluation with a plan for matched-relative allogeneic stem cell transplantation. After completing her regimen, a repeat bone marrow biopsy showed complete remission with hypocellular features and was negative for any residual leukemia with a normal female karyotype and FISH staining. Her physical deconditioning from her hospitalization improved with home rehabilitation, and she was readmitted 1.5 months after her HHS hospitalization for stem cell transplantation. She received high-dose conditioning chemotherapy with fludarabine and melphalan, followed by successful allogeneic stem cell transplantation, with her transplantation hospitalization complicated by mucositis, chemotherapy-related nausea and diarrhea, and neutropenic fever of unknown origin. Throughout her transplantation hospitalization, she has not experienced any derangements in glucose metabolism. As of the writing of this case, the patient is on posttransplant day 60 and in complete remission.

## Discussion

This case highlights a rare, previously unreported combination of PEG-asparaginase toxicities, including asparaginase-associated pancreatitis (AAP), HHS, and hypertriglyceridemia, in a young adult woman receiving induction treatment for ALL.

The expansion of the use of asparaginase in the treatment of adult ALL was initiated after studies showed that treatment with pediatric ALL regimens, including asparaginase, resulted in increased survival in AYA and adult patient populations with ALL (3–5). While asparaginase use has improved survival outcomes in adults, its expanded use also introduced the risk of unique toxicities such as AAP, endocrine dysfunction, and coagulopathy, as well as more common toxicities such as hyperglycemia and hepatotoxicity (15, 16). Asparaginase exhibits a similar toxicity profile in AYA/adult patients as in pediatric patients; however, pediatric patients experience lower rates of toxicity than their adult counterparts (**Table 2**) (17, 18).

**Table 2:** Comparison of toxicity rates between pediatric and AYA/adult patients receiving asparaginase treatment.

	Rate	Treatment
Pediatric asparaginase toxicity profile		
Hypersensitivity	10%–15%	• Discontinue infusion • Steroids, antihistamines, acetaminophen, and/or epinephrine • Consider premedication
Hepatotoxicity	Up to 60%	• Hold therapy if high-grade (grade 4) toxicity occurs; resume asparaginase with a resolution of hepatotoxicity
Pancreatitis	5%–10%	• Discontinue therapy if clinical pancreatitis occurs • Hold therapy for chemical pancreatitis; resume if no clinical symptoms present
Thromboembolism (TE)	5%–35%	• Low molecular weight heparin or unfractionated heparin if renal impairment present • Discontinue asparaginase if CNS or life-threatening TE occurs
Hyperlipidemia	11%–47%	• No treatment is required; asparaginase-related HTG does not confer an increased risk of AAP
Hyperglycemia	6%–25%	• Insulin; hold steroids and asparaginase for grades 3–4 hyperglycemia
Adult/AYA asparaginase toxicity profile		
Hypersensitivity	6%–21%	• Discontinue infusion • Steroids, antihistamines, acetaminophen, and/or epinephrine • Consider premedication
Hepatotoxicity	45%–90%	• Hold therapy if high-grade (grade 4) toxicity occurs; resume asparaginase with a resolution of hepatotoxicity
Pancreatitis	5%–14%	• Discontinue therapy if clinical pancreatitis occurs • Hold therapy for chemical pancreatitis; resume if no clinical symptoms present
TE	5%–41%	• Low molecular weight heparin or unfractionated heparin if renal impairment present • Discontinue asparaginase if CNS or life-threatening TE occurs
Hyperlipidemia	11%–50%	• No treatment is required; asparaginase-related HTG does not confer an increased risk of AAP
Hyperglycemia	30%–40%	• Insulin; hold steroids and asparaginase for grades 3–4 hyperglycemia

AYA, adolescent and young adult; CNS, central nervous system; HTG, hypertriglyceridemia; AAP, asparaginase-associated pancreatitis.

The incidence of asparaginase-associated hyperglycemia, hypertriglyceridemia, and AAP, as our patient experienced them, is estimated to be about 30%–40%, 50%, and 14%, respectively, in studies involving adult patients (Riley, Schlefman et al., 2021, Juluri, Siu et al., 2022). These toxicity rates are overall consistent, regardless of the asparaginase formulation that a patient receives. A retrospective analysis by Christ et al. in 2017 found that there were no significant differences in the rates of hepatotoxicity, AAP, thrombosis, or any other grades 3–4 toxicity in adults with ALL who received l-asparaginase *vs*. PEG-asparaginase (19).

The mechanisms of asparaginase-related toxicities are only starting to be understood and represent areas of current investigation. Studies exploring asparaginase-associated hyperglycemia suggest that asparagine depletion results in a corresponding decrease in pancreatic insulin production and additional resultant hyperglycemia (20, 21). Asparaginase-associated hypertriglyceridemia is likely related to increased very-low-density lipoprotein synthesis and decreased lipoprotein lipase activity levels (22). Interestingly, a case was recently reported involving a patient who experienced asparaginase-related hypertriglyceridemia and underwent subsequent genetic testing. The patient was a 28-year-old male with normal triglyceride levels at baseline who was diagnosed with ALL and treated with PEG-asparaginase, glucocorticoids, and chemotherapy. During his treatment, he experienced hypertriglyceridemia with a maximum level of 1793 mg/dl after PEG-asparaginase treatment. He underwent genetic analysis to evaluate for potential genetic variants contributing to susceptibility to derangement in triglyceride metabolism and was found to have a c.11G > A-p.(Arg4Gln) mutation in the APOC3 gene, which encodes a protein found in VLDL and chylomicrons, suggesting the potential for a variety of genetic predispositions to various asparaginase toxicities (23).

Studies investigating AAP have suggested two possible mechanisms of pancreatitis, including a variety of plasma amino acid level imbalances that persist for about 2 weeks after administration of asparaginase and impaired pancreatic acinar cell enzyme transfer resulting in cellular zymogen retention and subsequent cellular vacuolization and injury from premature enzyme activation (24, 25). Of note, hypertriglyceridemia does not correlate with or increase the risk of AAP, unlike its role as a cause of traditional non-asparaginase-related pancreatitis (26). Further understanding of the mechanisms underlying asparaginase-related toxicities will allow for more effective screening and interventions in patients receiving treatment for ALL.

To our knowledge, based on an extensive PubMed search, our case involves a previously unreported combination of asparaginase toxicities: AAP and HHS. Only one other case of asparaginase-associated HSS has been reported, occurring in a 10-year-old girl receiving treatment for ALL with l-asparaginase and prednisolone, and it occurred in the absence of AAP (9). Asparaginase has been shown to cause other serious endocrine dysfunctions, including diabetic ketoacidosis (DKA), and case reports of isolated DKA and DKA, in addition to AAP, have been previously published (10–13). The rate of asparaginase-related DKA is estimated to be about 0.8% based on a retrospective analysis of pediatric patients receiving treatment for ALL by Roberson et al. at St Jude Children’s Research Hospital (27). Mechanistically, endocrine and metabolic dysfunction caused by asparaginase are most likely due to its aforementioned effect on insulin production (20, 21). This, coupled with insulin resistance from glucocorticoids used in most ALL regimens, opens the possibility for glucose metabolism derangements ranging from mild hyperglycemia to diabetic ketoacidosis or HHS.

While DKA and especially HHS are rare potential toxicities, they are associated with high mortality risk, with HHS having an associated mortality rate of nearly 10%–20% (28). Risk factors for asparaginase-associated hyperglycemia include age >10 years old, obesity, and family history of diabetes, while asparaginase-associated DKA risk increases with increased age and hyperglycemia due to asparaginase (21, 27). It is reasonable to expect that similar risk factors also are associated with the risk of experiencing asparaginase-associated HHS as each condition shares similar underlying pathologic mechanisms, but further study is required to definitively identify HHS risk factors. Patients experiencing asparaginase-associated DKA are able to successfully complete asparaginase treatment without increased risk of recurrence, so it is reasonable to recommend continuation of asparaginase treatment in individuals that experience HHS after glucose control is achieved (27).

Our patient additionally experienced AAP, which represents a relatively common but severe asparaginase toxicity. AAP represents the most common reason for the discontinuation of asparaginase in the treatment of ALL (29). Studies have shown that the rate of secondary AAP upon rechallenge with asparaginase after a primary AAP episode ranges around 45%, and the European Society of Medical Oncology recommends that asparaginase should be permanently discontinued in adults that experience pancreatitis due to the high recurrence rate and associated mortality (30–33). Risk factors for AAP include having Native American ancestry, getting older, and increasing asparaginase dosing (34). Interestingly, even though asparaginase can cause elevations in triglycerides, hypertriglyceridemia does not represent a risk factor for AAP, unlike in nonmalignant pancreatitis, and there is no association between hypertriglyceridemia and AAP (26, 34). Additionally, AAP is strictly a clinical diagnosis, as elevations in pancreatic enzymes such as amylase or lipase do not predict a clinical occurrence of AAP (33). As such, asparaginase discontinuation is not recommended if pancreatic enzymes are elevated in the absence of other clinical findings suggestive of pancreatitis (29, 35).

In our patient’s case, she experienced AAP in addition to HHS and had to discontinue her asparaginase treatment component due to her pancreatitis. The connection between endocrine dysfunction such as DKA/HHS and pancreatitis has been well characterized, highlighting the potential that our patient’s HHS and AAP occurred together due to pancreatic dysfunction resulting in glucose metabolism derangements (36, 37). Patients receiving asparaginase should be educated to be vigilant for potential symptoms of acute pancreatitis so that they can quickly seek evaluation and care to prevent morbidity and mortality related to AAP. There are no studies to our knowledge comparing the monitoring of patients for asparaginase toxicity in an outpatient *vs*. inpatient setting, and consensus recommendations for asparaginase toxicity monitoring generally apply in the outpatient setting only. Examples of AAP monitoring recommendations include establishing baseline amylase/lipase levels before administering asparaginase and then rechecking levels 2–3 days postadministration and then weekly for at least 4 weeks (38). However, patient education on the signs and symptoms of AAP should remain paramount, as treatment discontinuation decisions should be made on the basis of clinical pancreatitis instead of a chemical diagnosis.

Unfortunately, discontinuation of asparaginase in patients who experience AAP results in poorer ALL survival outcomes (39, 40). For this reason, potential prophylactic measures to prevent pancreatitis in patients receiving asparaginase are being explored. The primary agent being explored for AAP prophylaxis is octreotide, with early data suggesting that treatment with octreotide in children experiencing AAP resulted in clinical improvement (41). A retrospective systematic review by Sakaguchi et al. using patient data from their center as well as previously published case reports found that the rate of secondary AAP in patients who received octreotide prophylaxis upon asparagine rechallenge was 36% (4/11 patients), which is nearly 10% lower than the established rechallenge AAP rate of 45% (42). However, the small sample size and methods of the review highlight the need for larger, more systematic studies to be conducted exploring octreotide use. Additionally, the potential benefits of improved survival with continued asparaginase treatment in ALL patients must be weighed in comparison to the remaining risk of AAP on rechallenge, which appears elevated even with octreotide prophylaxis.

Additionally, because AAP risk has been shown to increase with increasing asparagine doses, developing an effective AAP risk profile and screening tools to identify patients at increased risk of AAP is appealing (34). Genetic screening studies have identified multiple genomic loci and genotypes of interest that are associated with increased risk of AAP. Genome-wide association studies of patients in the NOPHO ALL2008 study by Wolthers et al. showed that increased risk for AAP was associated with single nucleotide polymorphisms (SNPs) in the *ULK2* gene on chromosome 17, with the rs281366 SNP showing the strongest risk association with AAP (30). *ULK2* polymorphisms involving EXON1: -493C>T and EXON1: -308C>G base changes were found to be significantly different between a group of AAP patients *vs*. a control group in a subsequent study, providing further evidence that *ULK2* plays a role in increasing the risk of AAP (40). An additional set of genes whose variations are associated with increased AAP risk are *PRSS1-PRSS2*, which encode trypsin; polymorphisms in these genes resulting in increased trypsinogen production were found to increase AAP risk (32). Further study into the genetic basis for AAP risk can help us not only risk stratify patients but also provide information to better identify the mechanisms of AAP and intervene directly in the pathologic process causing AAP.

## Conclusion

Overall, this case reports a previously unreported toxicity combination of pancreatitis and HSS secondary to asparaginase treatment for a patient with ALL. It also highlights the potential risks of asparaginase administration and the necessity for close monitoring of all patients receiving asparaginase. While incredibly rare, HHS due to asparaginase can have potentially severe consequences, and thus glucose levels should be closely monitored in patients receiving asparaginase. Additionally, while AAP is much more common than endocrine derangements such as DKA or HHS in patients receiving asparaginase, clinicians should monitor patients presenting with nonspecific clinical findings to ensure that patients receive proper treatment in the event that a patient presents with pancreatitis in addition to an underlying endocrine pathology.

## Data availability statement

The raw data supporting the conclusions of this article will be made available by the authors, without undue reservation.

## Ethics statement

Ethical review and approval was not required for the study on human participants in accordance with the local legislation and institutional requirements. The patients/participants provided their written informed consent to participate in this study.

## Author contributions

All listed authors have made a substantial contribution to the concept and/or design of the article, data analysis, and interpretation of data for the article. All authors contributed to the manuscript draft and/or revised it critically for important intellectual content; and approved the version to be published.
